# Characterizing the Clinical, Vascular, and Functional Phenotype of Metabolic Acidosis in Kidney Transplantation: A Cross-Sectional Study

**DOI:** 10.3390/jcm15052052

**Published:** 2026-03-08

**Authors:** Lucian Siriteanu, Adrian Covic, Cezar Băluță, Călin Namolovan, Simona Mihaela Hogaș, Irina Draga Căruntu, Luminița Voroneanu

**Affiliations:** 1 Department of Nephrology, Grigore T. Popa University of Medicine and Pharmacy, 700115 Iasi, Romania; siriteanulucian@gmail.com (L.S.); baluta_cezar@yahoo.com (C.B.); simonamihaelahogas@gmail.com (S.M.H.); irinadragacaruntu@gmail.com (I.D.C.); lumivoro@yahoo.com (L.V.); 2 “Dr. C. I. Parhon” University Hospital, 700503 Iasi, Romania; calinnamolovan67@gmail.com

**Keywords:** kidney transplantation, metabolic acidosis, bicarbonate, pulse wave velocity, arterial stiffness, frailty, inflammation, graft function

## Abstract

**Introduction**: Metabolic acidosis is common after kidney transplantation and is associated with adverse outcomes. However, its vascular and functional correlates in kidney transplant recipients remain insufficiently characterized. **Methods:** We conducted a cross-sectional study of adult kidney transplant recipients attending routine outpatient visits at a tertiary transplant center. Metabolic acidosis was defined as serum bicarbonate < 22 mmol/L. Arterial stiffness was assessed by carotid–femoral pulse wave velocity (PWV), and physical frailty was evaluated using the Fried frailty phenotype. Multivariable regression models were used to identify determinants of metabolic acidosis and to examine its association with arterial stiffness and frailty severity. **Results:** Among 239 patients (median age 46 years), 154 (64%) had metabolic acidosis. Lower estimated glomerular filtration rate and higher systemic inflammation were independently associated with metabolic acidosis. Metabolic acidosis was independently associated with higher arterial stiffness, with a 1.41 m/s higher PWV after adjustment for age, sex, blood pressure, kidney function, and diabetes mellitus (*p* < 0.001). Although metabolic acidosis was associated with greater frailty severity in minimally adjusted models, this association was attenuated and no longer statistically significant after further adjustment for kidney function, diabetes, and inflammation. In stable kidney transplant recipients, metabolic acidosis is independently associated with increased arterial stiffness but not with frailty after accounting for key clinical confounders. **Conclusions:** These findings highlight metabolic acidosis as a marker of vascular vulnerability and a potential therapeutic target after kidney transplantation.

## 1. Introduction

Metabolic acidosis is a frequent complication after kidney transplantation, with reported prevalences reaching up to 70% and an earlier onset than in non-transplanted patients with chronic kidney disease [1,2]. Traditionally, metabolic acidosis has been considered a graft-related condition, resulting from a reduction in functional nephron mass that impairs bicarbonate reabsorption and ammoniagenesis. However, in kidney transplant recipients, additional transplant-specific factors have been implicated, including immunosupressants and immunologically mediated changes associated with rejection [3,4]. Metabolic acidosis is clinically relevant, as it has been consistently associated with poorer graft survival, increased cardiovascular events, and higher mortality in both observational and registry studies [3,5,6].

Although renal outcomes have been the primary focus of research, metabolic acidosis in chronic kidney disease has been linked to broader systemic consequences, including accelerated vascular aging and physical decline. However, in kidney transplant recipients, the integrated clinical, vascular, and functional phenotype associated with metabolic acidosis remains incompletely characterized. Specifically, arterial stiffness—a key marker of vascular aging—has not been systematically evaluated in relation to acid-base status in this population, nor has physical frailty been examined as a potential correlate of metabolic acidosis after transplantation.

Arterial stiffness, assessed by carotid-femoral pulse wave velocity (PWV), provides an objective measure of vascular aging with established clinical utility. In kidney transplant recipients, elevated PWV is a strong and independent predictor of mortality, cardiovascular events, and progressive graft dysfunction, supporting its role as a marker for long-term risk stratification. However, its relationship with metabolic acidosis—a potentially modifiable factor—has not been systematically explored in this population [7].

Similarly, physical frailty—assessed using the validated Fried phenotype—represents a complementary dimension of biological vulnerability that has not been evaluated in relation to metabolic acidosis in transplant recipients. Frailty is prevalent among kidney transplant recipients (approximately 14%) and is associated with an approximately twofold higher risk of mortality [8,9].

Comprehensive characterization of the metabolic acidosis phenotype requires assessment of both structural vascular changes and their functional consequences. PWV and the Fried phenotype provide complementary, objective tools for this integrated assessment [10,11,12,13].

In the present study, we use the term phenotype to refer to the integrated clinical, vascular, and functional expression of metabolic acidosis, encompassing biochemical abnormalities, vascular alterations assessed by arterial stiffness, and functional impairment assessed by physical frailty.

Therefore, the primary aim of this study was to characterize the clinical, vascular, and functional manifestations associated with metabolic acidosis in stable kidney transplant recipients through contemporaneous assessment of arterial stiffness by carotid–femoral pulse wave velocity and physical frailty using the Fried phenotype.

## 2. Materials and Methods

### 2.1. Study Design and Population

Adult kidney transplant recipients (≥18 years) attending routine outpatient visits at a tertiary referral renal transplant center in Iași, Romania, between February and May 2024 were screened for eligibility.

Inclusion criteria were: (1) kidney transplantation (living or deceased donor) performed ≥3 months prior to assessment; (2) stable graft function, defined as an estimated glomerular filtration rate (eGFR) ≥ 15 mL/min/1.73 m^2^ calculated using the creatinine-based CKD-EPI equation, <20% variation in serum creatinine over the preceding 6 months, and no recent biopsy-proven or clinically treated acute rejection; (3) stable immunosuppressive regimen without major changes (drug class modification or >25% dose adjustment) in the preceding 3 months; and (4) ability to complete pulse wave velocity and frailty assessments.

Exclusion criteria included: (1) acute medical events within 3 months prior to assessment, such as acute rejection, hospitalization for infection or cardiovascular events, or acute gastrointestinal illness; (2) severe comorbidities independently affecting acid–base balance, including decompensated heart failure (New York Heart Association class IV), severe chronic obstructive pulmonary disease with documented hypercapnia (PaCO_2_ > 50 mmHg), liver cirrhosis, or poorly controlled diabetes mellitus with recent ketoacidosis; (3) current use of medications known to directly affect acid–base status (high-dose loop diuretics > 80 mg/day furosemide equivalent or carbonic anhydrase inhibitors); (4) technical limitations precluding pulse wave velocity measurement (atrial fibrillation with irregular rhythm, severe peripheral arterial disease, or inability to lie supine); (5) inability to perform frailty assessment due to severe mobility or cognitive impairment; or (6) incomplete data for primary exposure or outcomes.

Between February 2024 and September 2024, 350 patients were assessed for eligibility. Of these, 83 were excluded at screening due to recent acute rejection, unstable graft function, intercurrent acute medical events, major changes in immunosuppressive regimen, severe concomitant diseases, current or recent alkali therapy, or refusal to provide informed consent. A total of 267 patients were eligible for confirmation and underwent reassessment of serum bicarbonate levels after four weeks.

At confirmation, 28 patients were excluded: 12 due to spontaneous normalization of serum bicarbonate levels and 16 due to acute intercurrent events or therapeutic modifications. The final analytical cohort comprised 239 patients.

Patients were not selected based on acid–base status; however, for analytical purposes, they were subsequently classified according to the presence (serum bicarbonate < 22 mmol/L) or absence (≥22 mmol/L) of metabolic acidosis.

### 2.2. Data Collection

All clinical, laboratory, vascular, and functional assessments were performed contemporaneously during a single index outpatient visit to ensure temporal alignment and minimize the influence of short-term metabolic fluctuations. To minimize the likelihood of transient or acute acid–base disturbances, serum bicarbonate was assessed on two separate occasions at least four weeks apart as part of routine outpatient follow-up. Only patients with concordant bicarbonate values meeting the study definition were included in the final analysis. In addition, all participants had stable graft function—defined by <20% variation in serum creatinine over the preceding 3 months and absence of recent acute medical events—supporting the interpretation of serum bicarbonate levels as reflecting chronic steady-state acid–base status rather than acute metabolic derangements.

Data were prospectively collected as part of routine clinical care and retrieved from institutional electronic medical records and the transplant database. Variables were predefined before analysis and grouped into clinically relevant domains.

Clinical and laboratory data

Demographic and transplant-related variables included age, sex, body mass index, time since transplantation, donor type and age, HLA mismatches, prior rejection episodes, anti-HLA antibody status, and current immunosuppressive regimen (calcineurin inhibitor type and trough levels, mycophenolate, corticosteroids). Laboratory measurements were performed in the central hospital laboratory using standardized methods. Renal function was assessed using the CKD-EPI (2021) estimated glomerular filtration rate (eGFR) equation. Serum bicarbonate, electrolytes, hemoglobin, and C-reactive protein were measured. Proteinuria was quantified using spot urine protein-to-creatinine ratio (UPCR) and analyzed after natural logarithmic transformation [ln(UPCR + 1)] to account for skewed distribution. Comorbidities were defined based on documented diagnoses and active treatment, with overall burden quantified using the Charlson Comorbidity Index.

Vascular assessment

Arterial stiffness: measured through applanation tonometry of the carotid and femoral arteries using the SphygmoCor system (AtCor Medical Pty Ltd., Westmead, NSW, Australia) according to international guidelines [14,15]. The protocol includes the patient preparation like resting for at least 10 min, measuring simultaneously the pulse wave of the carotid and femoral arteries, the transit time from the R-wave of the electrocardiogram to the foot of the carotid-femoral pulse, and the transit time measured between the feet of the two waveforms; the carotid-femoral PWV is calculated by dividing the difference between these 2 transit times by the time it takes to travel the arterial path length.

Frailty assessment

Physical frailty was assessed using the Fried frailty phenotype, comprising five components: unintentional weight loss (≥4.5 kg in past year), self-reported exhaustion (CES-D questions), low physical activity (IPAQ short form), slow gait speed (4-m walk test), and weak grip strength measured using Jamar hydraulic dynamometer (Patterson Medical, Warrenville, IL, USA) with maximum of three measurements, sex- and BMI-adjusted thresholds [16]. Participants were classified as robust (0 components), pre-frail (1–2 components), or frail (≥3 components), analyzed as both categorical and ordinal variables.

### 2.3. Statistical Analysis

Baseline characteristics were summarized for the overall cohort and stratified by the presence or absence of metabolic acidosis. Continuous variables are presented as median (interquartile range), and categorical variables as number (percentage). Between-group differences were quantified using standardized mean differences (SMD), which provide a scale-independent measure of imbalance and are not influenced by sample size. For continuous variables, SMD was calculated as the difference in means divided by the pooled standard deviation. For binary and categorical variables, SMDs were computed from differences in proportions. For ordinal variables, SMDs were derived from standardized differences across category distributions. An absolute SMD value > 0.10 was considered indicative of a potentially meaningful between-group imbalance. SMDs were used to describe the magnitude of between-group differences rather than to infer statistical significance.

To identify clinical and biological factors independently associated with metabolic acidosis at baseline, multivariable logistic regression analyses were performed with metabolic acidosis as the dependent variable. Given the relatively small sample size and the presence of covariates with low event frequencies, penalized logistic regression with Firth’s correction was used to reduce small-sample bias and instability of maximum likelihood estimates in models with multiple covariates. Covariates were selected a priori based on clinical relevance and prior literature and included recipient age, sex, estimated glomerular filtration rate, diabetes mellitus, donor age, proteinuria, anti-HLA antibodies, systemic inflammation assessed by log-transformed C-reactive protein, and RAAS blockade. Results are presented as odds ratios with 95% confidence intervals. Multicollinearity was assessed using variance inflation factors. As a complementary analysis, serum bicarbonate (mmol/L) was modeled as a continuous outcome using multivariable linear regression with the same covariates as in the logistic regression model. Robust (HC3) standard errors were used to account for potential heteroskedasticity.

Arterial stiffness was assessed by carotid–femoral pulse wave velocity (PWV, m/s). To evaluate the cross-sectional association between metabolic acidosis and arterial stiffness, PWV was modeled as a continuous outcome using multivariable linear regression, with metabolic acidosis (bicarbonate < 22 mmol/L) as the exposure. Mean arterial pressure (MAP) was calculated as DBP + (SBP − DBP)/3 and included for hemodynamic adjustment. Two models were prespecified: a core model adjusted for age, sex, and MAP, and a fully adjusted model that also included eGFR and diabetes. Robust (HC3) standard errors were used.

Frailty was assessed at the index outpatient visit using the Fried frailty phenotype and categorized as robust (0 criteria), pre-frail (1–2 criteria), or frail (≥3 criteria). Frailty severity was analyzed as an ordered categorical outcome. Associations between metabolic acidosis and frailty severity were examined using ordinal logistic regression with proportional odds. Metabolic acidosis, defined as a serum bicarbonate concentration < 22 mmol/L, was the exposure of interest. Two prespecified models were fitted: a minimally adjusted model including age and sex, and a fully adjusted model additionally including estimated glomerular filtration rate, diabetes mellitus, and systemic inflammation assessed by log-transformed C-reactive protein. The proportional odds assumption was assessed and was not materially violated. Individual components of the Fried frailty phenotype were not included as covariates to avoid overadjustment and collinearity. Covariates were selected a priori based on biological plausibility and prior literature. Results are reported as odds ratios with 95% confidence intervals. All analyses were restricted to baseline data. Given the cross-sectional design, analyses were intended to characterize associations rather than infer causality.

All statistical analyses were performed using R software (R Foundation for Statistical Computing, Vienna, Austria) in RStudio version 2026.01.0 (Posit Software, PBC, Boston, MA, USA).

### 2.4. Ethical Consideration

The study was conducted in accordance with the Declaration of Helsinki and approved by the Institutional Ethics Committee of “Dr. C. I. Parhon” Hospital and “Grigore T. Popa” University of Medicine and Pharmacy, Iași (approval no. 1796/27.02.2024).

## 3. Results

### 3.1. Baseline Characteristics

Baseline demographic, transplant-related, clinical, and laboratory characteristics stratified by metabolic acidosis status are shown in Table 1. Between-group differences are presented as standardized mean differences (SMDs) to quantify the magnitude of imbalance rather than statistical significance. Age, sex distribution, and body mass index were broadly similar between groups (|SMD| < 0.15).

In contrast, marked differences were observed in transplant-related and metabolic domains. Patients with metabolic acidosis had lower graft function (median eGFR 47 vs. 66 mL/min/1.73 m^2^) and a higher burden of proteinuria, alongside a higher immunological risk profile, including a greater prevalence of anti-HLA antibodies (51% vs. 28%) and greater HLA mismatching.

The metabolic acidosis group also exhibited a distinct biochemical and inflammatory profile, characterized by higher C-reactive protein levels (median 5.6 vs. 3.1 mg/L), lower hemoglobin, and differences in electrolyte parameters (lower sodium and higher chloride concentrations).

Comorbidity burden was higher among patients with metabolic acidosis, driven primarily by higher rates of diabetes and cardiovascular disease, resulting in a higher Charlson Comorbidity Index. Importantly, vascular and functional assessments showed substantially higher arterial stiffness (median PWV 9.9 vs. 8.1 m/s) and a higher frailty burden (frailty prevalence 16.9% vs. 5.9%) in the metabolic acidosis group.

Given the cross-sectional design, these findings represent contemporaneous associations and should not be interpreted as causal effects.

### 3.2. Clinical and Biochemical Correlates of Metabolic Acidosis

In fully adjusted analyses, lower estimated glomerular filtration rate (eGFR) and higher systemic inflammation were independently associated with metabolic acidosis. Each 1 mL/min/1.73 m^2^ higher eGFR was associated with lower odds of metabolic acidosis (odds ratio [OR] 0.98, 95% confidence interval [CI] 0.96–0.99; *p* = 0.002), whereas higher log-transformed C-reactive protein was strongly associated with metabolic acidosis (OR 3.67 per log [1+CRP] unit, 95% CI 1.93–7.26; *p* < 0.001). Recipient age was inversely associated with metabolic acidosis (OR 0.96 per year, 95% CI 0.93–0.99; *p* = 0.007).

**Figure 1:**
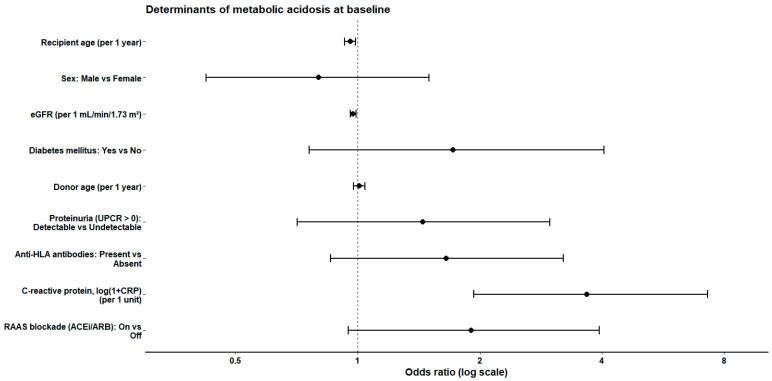
Forrest plot displaying adjusted odds ratios (log scale) and 95% confidence intervals derived from multivariable penalized (Firth) logistic regression. Metabolic acidosis was defined as serum bicarbonate < 22 mmol/L. The model was adjusted for recipient age, sex, estimated glomerular filtration rate, diabetes mellitus, donor age, proteinuria, anti-HLA antibodies, systemic inflammation (log-transformed C-reactive protein), and RAAS blockade. Odds ratios are presented on a logarithmic scale.

Sex, diabetes mellitus, donor age, proteinuria, anti-HLA antibody status, and renin–angiotensin–aldosterone system (RAAS) blockade were not independently associated with metabolic acidosis after multivariable adjustment (Table 2, Figure 1). Results were robust across sensitivity analyses using alternative acidosis definitions and model specifications (Supplementary Tables S1–S4).

In complementary analyses modeling serum bicarbonate as a continuous outcome, higher eGFR was independently associated with higher bicarbonate levels, whereas higher systemic inflammation, detectable proteinuria, and RAAS blockade were associated with lower bicarbonate concentrations, consistent with the logistic regression findings (Supplementary Table S5).

### 3.3. Association Between Metabolic Acidosis and Arterial Stiffness

**Figure 2:**
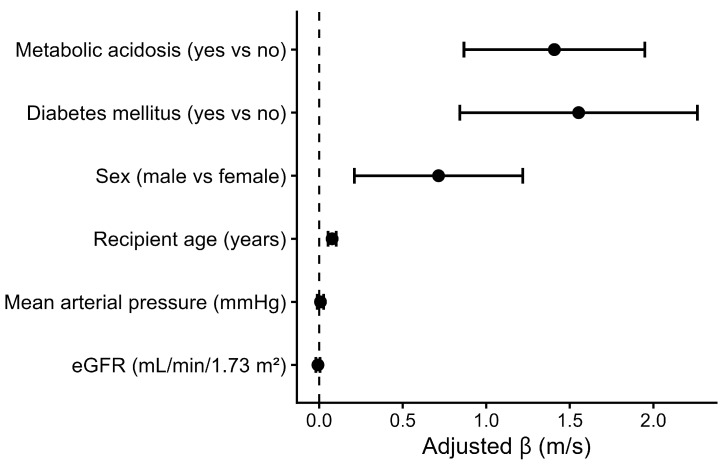
Independent association between metabolic acidosis and arterial stiffness. Forrest plot showing adjusted regression coefficients (β) and 95% confidence intervals for pulse wave velocity, derived from the multivariable linear regression model. Models were adjusted a priori for age, sex, mean blood pressure, renal function and diabetes mellitus.

In multivariable linear regression, metabolic acidosis was independently associated with higher arterial stiffness. After adjustment for age, sex, and mean arterial pressure, metabolic acidosis was associated with a 1.73 m/s higher PWV (β = 1.73; 95% CI 1.19–2.27; *p* < 0.001), an association that remained significant after additional adjustment for eGFR and diabetes mellitus (β = 1.41 m/s; 95% CI 0.87–1.95; *p* < 0.001). Older age, male sex, and diabetes mellitus were also independently associated with higher PWV, whereas mean arterial pressure and eGFR were not (Table 3, Figure 2).

### 3.4. Association Between Metabolic Acidosis and Frailty

**Figure 3:**
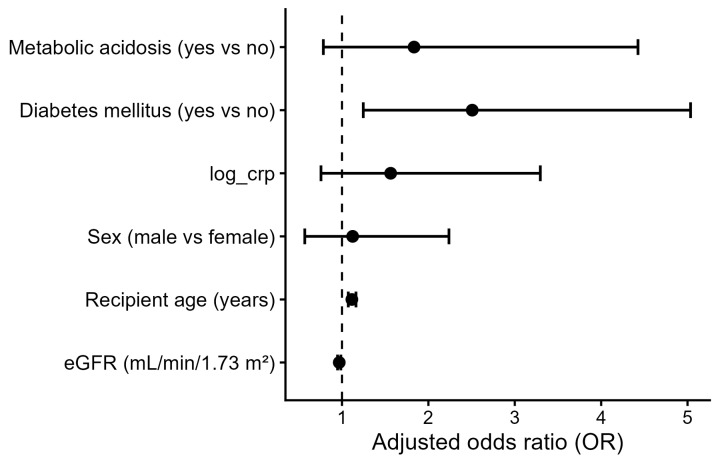
Association between metabolic acidosis and frailty severity. Forest plot showing odds ratios (ORs) and 95% confidence intervals for the association between metabolic acidosis and frailty severity, assessed using ordinal logistic regression models. Frailty severity was defined according to the Fried frailty phenotype. Model 1 was adjusted for recipient age and sex, while Model 2 was additionally adjusted for kidney function (eGFR), diabetes mellitus, and systemic inflammation (log-transformed C-reactive protein). ORs greater than 1 indicate higher odds of being in a more severe frailty category.

In ordinal logistic regression adjusted for age and sex, metabolic acidosis was associated with greater frailty severity (OR 4.51, 95% CI 2.21–9.79; *p* < 0.001). After additional adjustment for kidney function, diabetes mellitus, and systemic inflammation, the association was attenuated and no longer statistically significant (OR 1.83, 95% CI 0.78–4.43; *p* = 0.167). In the fully adjusted model, older age, lower eGFR, and diabetes mellitus remained independently associated with higher frailty severity (Table 4, Figure 3).

## 4. Discussion

In this cross-sectional study of stable kidney transplant recipients, multivariable analyses showed that metabolic acidosis was primarily driven by impaired kidney function and systemic inflammation, was independently associated with increased arterial stiffness, and was not independently associated with frailty after comprehensive adjustment for major clinical confounders. The observed association between RAAS blockade and lower bicarbonate levels in complementary analyses should be interpreted with caution. From a pathophysiological perspective, renin–angiotensin–aldosterone system inhibition may contribute to impaired distal acidification through reduced aldosterone-mediated hydrogen ion secretion. However, in clinical practice, RAAS inhibitors are preferentially prescribed in patients with proteinuria or graft dysfunction—conditions independently associated with impaired renal acid excretion. Therefore, this association may reflect confounding by indication rather than a direct causal contribution of RAAS blockade to metabolic acidosis.

Arterial stiffness is highly prevalent across all stages of chronic kidney disease and persists after kidney transplantation, being independently associated with lower estimated glomerular filtration rate and supporting a bidirectional cardio–renal relationship. Chronic kidney disease–related vascular calcification, inflammation, and endothelial dysfunction promote arterial stiffening, which in turn accelerates cardiac overload and renal function decline through enhanced pulsatile pressure transmission, thereby perpetuating a vicious cardio–renal cycle even after transplantation [17,18]. Interestingly, despite the marked between-group difference in eGFR observed at baseline, kidney function was not independently associated with arterial stiffness in the fully adjusted PWV model. This finding may reflect the relatively narrow eGFR distribution in this cohort or the influence of transplant-specific vascular determinants that may persist independently of current graft function.

Beyond eGFR, most of the implicated risk factors include older age, dyslipidemia, diabetes mellitus, and mean blood pressure levels; however, in recent years, increasing attention has also been directed toward non-traditional risk factors [19]. Observational studies report inconsistent associations between metabolic acidosis and arterial stiffness: an independent relationship is observed in patients with chronic kidney disease (KNOW-CKD), but not in community-dwelling older adults after adjustment for renal function (Health ABC), suggesting that the vascular impact of acidosis may be more relevant in settings of impaired kidney function, such as kidney transplantation [20,21]. However, data in kidney transplant recipients have been lacking. In this context, our study demonstrates that metabolic acidosis is independently associated with increased arterial stiffness in stable kidney transplant recipients, even after adjustment for age, blood pressure, kidney function, and diabetes. In kidney transplant recipients, the association between metabolic acidosis and arterial stiffness may persist independently of eGFR because transplantation represents a distinct pathophysiological state characterized by prior cumulative uremic exposure, chronic immune activation, and immunosuppressive therapy, all of which may amplify vascular vulnerability to acid–base disturbances. By contrast, in non-transplant CKD populations, adjustment for eGFR may capture much of the acidosis-related vascular risk, thereby attenuating the observed association.

In contrast to arterial stiffness, the association between metabolic acidosis and frailty was substantially attenuated after multivariable adjustment. In minimally adjusted models, metabolic acidosis was associated with greater frailty severity; however, this association was no longer statistically significant after accounting for estimated glomerular filtration rate, diabetes mellitus, and systemic inflammation. This pattern suggests that metabolic acidosis does not act as an independent driver of frailty but rather reflects the burden of underlying kidney dysfunction and systemic disease. Frailty is a multifactorial construct integrating metabolic, inflammatory, and comorbidity-related pathways, and the strong independent associations observed for lower kidney function and diabetes in the fully adjusted model support their central role in determining frailty severity. Consistent with this interpretation, systemic inflammation was associated with metabolic acidosis but did not independently predict frailty after adjustment, indicating that acid–base disturbances may serve as a marker of biological vulnerability rather than a direct determinant of functional decline in kidney transplant recipient. The attenuation of the association between systemic inflammation and frailty under stricter definitions of metabolic acidosis in sensitivity analyses may reflect small-sample effects or biological heterogeneity within the frailty phenotype, which integrates multiple metabolic and functional pathways not exclusively driven by acid–base disturbances.

Frailty has a negative impact on both mortality and quality of life in kidney transplant recipients [22]. Frailty prevalence increases with advancing stages of chronic kidney disease, with estimated glomerular filtration rate consistently reported as an independent predictor in the literature [23]. From a pathophysiological perspective, metabolic acidosis is closely associated with sarcopenia, a core component of the Fried frailty phenotype. Moreover, available data suggest that metabolic acidosis has been associated with increased inflammatory signaling and muscle catabolism in experimental and clinical settings, thereby sustaining systemic inflammation, reducing protein intake, and contributing to secondary sarcopenia [24].

Taken together, these findings suggest that metabolic acidosis may preferentially reflect vascular rather than functional vulnerability in kidney transplant recipients. Arterial stiffness assessed by carotid–femoral pulse wave velocity captures subclinical vascular damage and early vascular aging, which may precede overt clinical or functional decline. In contrast, physical frailty represents a later, integrative manifestation of cumulative systemic disease, comorbidity burden, and reduced physiological reserve. This temporal and conceptual distinction may explain why metabolic acidosis remained independently associated with arterial stiffness but not with frailty after comprehensive adjustment, highlighting differential pathways linking acid–base disturbances to vascular and functional outcomes after transplantation.

From a clinical perspective, metabolic acidosis may represent an easily measurable marker associated with increased vascular risk after kidney transplantation. Serum bicarbonate is routinely assessed in clinical practice and may provide additional information beyond traditional cardiovascular risk factors and kidney function alone. Importantly, metabolic acidosis represents a potentially modifiable condition; however, the present findings should not be interpreted as supporting therapeutic intervention. Rather, they underscore the need for further investigation into whether correction of acid–base disturbances may influence vascular health in kidney transplant recipients.

Longitudinal and interventional studies are needed to determine whether correction of metabolic acidosis can attenuate vascular aging and improve long-term cardiovascular and graft outcomes after kidney transplantation. Future research should focus on prospective evaluation of arterial stiffness trajectories and on randomized trials assessing the vascular impact of alkali therapy in this population.

## 5. Limitations

This study has several limitations that should be acknowledged. First, the cross-sectional design precludes causal inference and limits the ability to determine the temporal direction of the observed associations between metabolic acidosis, arterial stiffness, and frailty.

Second, although comprehensive multivariable adjustment was performed, residual confounding cannot be excluded. Certain factors relevant to vascular and functional outcomes—such as markers of mineral and bone metabolism (e.g., calcium, phosphorus, parathyroid hormone), nutritional status, body composition or sarcopenia-related measures, as well as time-updated immunosuppressive exposure (e.g., calcineurin inhibitor trough levels), nutritional status, body composition, and inflammatory mediators beyond C-reactive protein—were not available and therefore not included in the analyses. Fourth, the study was conducted at a single tertiary transplant center, which may limit the generalizability of the findings to other transplant populations. Information regarding pre-emptive transplantation status or dialysis vintage prior to transplantation was not systematically available and therefore could not be accounted for in the analysis.

In addition, the relatively young median age of the study population (46 years), compared with many transplant cohorts, may further limit the external generalizability of these findings.

Finally, the modest sample size, particularly in the subgroup of frail patients, may have limited statistical power and increased the risk of type II error in fully adjusted models to detect weaker associations. Nevertheless, the use of standardized, contemporaneous vascular and functional assessments and the consistency of findings across multiple modeling approaches support the robustness of the main results.

## 6. Conclusions

In stable kidney transplant recipients, metabolic acidosis is independently associated with higher arterial stiffness in cross-sectional analyses but not with frailty after accounting for kidney function, diabetes, and systemic inflammation. These findings support metabolic acidosis as a marker of vascular vulnerability after transplantation and highlight the need for longitudinal and interventional studies to determine whether correction of acid–base disturbances can improve vascular and long-term clinical outcomes.

## Figures and Tables

**Table 1:** Baseline demographic, clinical, and laboratory characteristics stratified by metabolic acidosis. Values are presented as median (interquartile range) or *n* (%). Differences are expressed as standardized mean differences (SMD).

Characteristic	Overall (*N* = 239)	No Metabolic Acidosis (*N* = 85)	Metabolic Acidosis (*N* = 154)	SMD
Demographic characteristics				
Age, years (median, IQR)	46 (38–54)	46 (39–55)	46 (38–54)	0.09
Male sex, *n* (%)	143 (60)	54 (64)	89 (58)	0.12
Body mass index, kg/m^2^	25.9 (22.5–28.3)	25.6 (22.6–27.5)	26.1 (22.5–29.1)	−0.13
Transplant-related characteristics				
Time since transplantation, months (median, IQR)	48 (16–101)	47 (20–85)	52 (16–111)	−0.19
Donor type	-	-	-	0.10
Deceased donor, *n* (%)	119 (50)	45 (53)	74 (48)	
Living donor, *n* (%)	120 (50.2)	40 (47.1)	80 (51.9)	
Donor age, years (median, IQR)	49 (43–56)	46 (42–53)	52 (43–56)	−0.23
HLA mismatches, total (median, IQR)	3 (2–3)	2 (2–3)	3 (2–3)	−0.44
Delayed graft function, *n* (%)	12 (5)	2 (2)	10 (6)	−0.20
Basiliximab induction, *n* (%)	223 (93)	82 (96)	141 (92)	0.21
Thymoglobulin induction, *n* (%)	16 (7)	2 (2)	14 (9)	−0.29
Tacrolimus use, *n* (%)	166 (69)	60 (71)	106 (69)	0.04
Cyclosporine use, *n* (%)	70 (29)	24 (28)	46 (30)	−0.04
Prednisone use, *n* (%)	209 (87)	75 (88)	134 (87)	0.04
Mycophenolate use, *n* (%)	230 (96)	79 (93)	151 (98)	−0.25
Anti-HLA antibodies, *n* (%)	102 (43)	24 (28)	78 (51)	−0.46
Baseline medication				
Loop diuretic (furosemide), *n* (%)	8 (3.3)	0 (0.0)	8 (5.2)	0.33
Mineralocorticoid receptor antagonists (spironolactone), *n* (%)	28 (11.7)	9 (10.6)	19 (12.3)	0.06
RAAS blockade (ACEi or ARB), *n* (%)	72 (30.1)	19 (22.6)	53 (34.4)	0.26
Renal function and laboratory parameters				
Serum bicarbonate, mmol/L (median, IQR)	21.0 (20.0–24.0)	25.0 (24.0–27.0)	20.0 (19.0–21.0)	2.95
eGFR, mL/min/1.73 m^2^ (median, IQR)	55 (40–70)	66 (53–80)	47 (35–62)	0.81
Serum creatinine, mg/dL (median, IQR)	1.45 (1.19–1.84)	1.22 (0.99–1.58)	1.60 (1.30–2.07)	−0.76
UPCR > 0 mg/g, *n* (%)	84 (35.1)	17 (20)	67 (43.5)	0.52
UPCR (mg/g) among patients with UPCR > 0, median (IQR)	-	470 (340–920)	1100 (500–1435)	-
Hemoglobin, g/dL (median, IQR)	12.1 (10.6–13.6)	13.3 (12.4–14.5)	11.4 (10.2–13.0)	0.71
C-reactive protein, mg/L (median, IQR)	4.5 (2.5–7.2)	3.1 (2.1–4.3)	5.6 (3.2–8.0)	−0.71
Serum sodium, mEq/L (median, IQR)	139.0 (137.0–140.0)	140.0 (138.0–141.0)	138.0 (137.0–140.0)	0.63
Serum potassium, mEq/L	4.30 (4.02–4.60)	4.20 (3.90–4.50)	4.30 (4.10–4.70)	−0.37
Serum chloride, mEq/L (median, IQR)	106.0 (104.0–108.0)	104.0 (103.0–107.0)	107.0 (104.0–109.0)	−0.61
Anion gap, mEq/L (median, IQR)	11.0 (9.0–12.0)	10.0 (8.0–11.0)	11.0 (10.0–13.0)	−0.67
Total proteins, g/dL (median, IQR)	7.1 (6.7–7.5)	7.2 (6.9–7.5)	7.1 (6.6–7.5)	0.27
Comorbidities				
Systolic blood pressure, mmHg (median, IQR)	140 (130–150)	143 (134–151)	139 (130–148)	0.15
Diastolic blood pressure, mmHg (median, IQR)	87 (78–95)	90 (80–97)	86 (76–93)	0.25
Hypertension, *n* (%)	197 (82)	70 (82)	127 (82)	−0.00
Diabetes mellitus, *n* (%)	54 (23)	13 (15)	41 (27)	−0.28
Coronary heart disease, *n* (%)	34 (14)	8 (9)	26 (17)	−0.22
Heart failure, *n* (%)	15 (6)	4 (5)	11 (7)	−0.10
Atrial fibrillation, *n* (%)	11 (5)	2 (2)	9 (6)	−0.18
Charlson Comorbidity Index, *n* (%)	-	-	-	−0.31
0–1	180 (75.3)	72 (84.7)	108 (70.1)	
2–3	34 (14.2)	9 (10.6)	25 (16.2)	
≥4	25 (10.5)	4 (4.7)	21 (13.6)	
Functional, vascular, and frailty-related measures				
Pulse wave velocity, m/s (median, IQR)	9.4 (7.6–11.2)	8.1 (6.9–9.7)	9.9 (8.5–11.7)	−0.68
Handgrip strength, kgf (median, IQR)	26.0 (22.0–29.0)	26.0 (22.0–31.0)	25.0 (22.0–28.0)	0.12
Frailty category (Fried), *n* (%)	-	-	-	−0.42
Robust	169 (70.7)	71 (83.5)	98 (63.6)	
Pre-frail	39 (16.3)	9 (10.6)	30 (19.5)	
Frail	31 (13)	5 (5.9)	26 (16.9)	

Notes. Values are presented as median (interquartile range) for continuous variables and as number (percentage) for categorical variables. Standardized mean differences (SMD) are used to quantify between-group differences; values are shown with a sign indicating the direction of the difference between groups. The magnitude (absolute value) of the SMD was used to assess between-group imbalance, with values > 0.10 considered meaningful. For continuous variables, SMDs were calculated using means and standard deviations derived from the underlying data, even when variables are summarized as medians (IQR) due to skewed distributions. For ordinal categorical variables, SMD was calculated using standardized differences based on category proportions. Positive SMD values indicate higher values in the metabolic acidosis group. The urine protein-to-creatinine ratio (UPCR) was expressed in mg/g. Because of a high proportion of zero values, UPCR was reported as the proportion of patients with UPCR > 0 and as the median (IQR) among those with detectable proteinuria. Charlson Comorbidity Index was categorized as 0–1, 2–3, and ≥4. ACEi: angiotensin-converting enzyme inhibitors; ARB: angiotensin receptor blockers; RAAS: renin–angiotensin–aldosterone system; UPCR: urine protein-to-creatinine ratio; PWV: pulse wave velocity; SMD: standardized mean difference.

**Table 2:** Clinical and biochemical factors independently associated with metabolic acidosis. Multivariable penalized (Firth) logistic regression was used to identify factors independently associated with metabolic acidosis, defined as serum bicarbonate < 22 mmol/L. Results are presented as odds ratios (ORs) with 95% confidence intervals (CIs). The model was adjusted a priori for recipient age, sex, estimated glomerular filtration rate (eGFR), diabetes mellitus, donor age, proteinuria, anti-HLA antibodies, systemic inflammation assessed by log-transformed C-reactive protein, and RAAS blockade. Penalized regression was applied to reduce small-sample bias. Multicollinearity was assessed using variance inflation factors.

Variable	Odds Ratio (95% CI)	*p* Value
Recipient age (years)	0.96 (0.93, 0.99)	0.007
Sex: Male vs. Female	0.80 (0.42, 1.50)	0.491
eGFR (mL/min/1.73 m^2^)	0.98 (0.96, 0.99)	0.002
Diabetes: Yes vs. No	1.72 (0.76, 4.04)	0.194
Donor age (years)	1.01 (0.98, 1.04)	0.540
Proteinuria: Detectable vs. Undetectable	1.45 (0.71, 2.98)	0.309
Anti-HLA: Present vs. Absent	1.65 (0.86, 3.21)	0.133
Log C-reactive protein [log(1+CRP)]	3.67 (1.93, 7.26)	<0.001
RAAS blockade: On vs. Off	1.90 (0.95, 3.93)	0.071

Odds ratios (ORs) with 95% confidence intervals are shown. Penalized logistic regression was used to reduce small-sample bias. All variance inflation factors were <2.

**Table 3:** Independent association between metabolic acidosis and arterial stiffness (pulse wave velocity) at baseline. Values represent regression coefficients (β) expressed as m/s with 95% confidence intervals, derived from multivariable linear regression. Covariates were selected a priori based on clinical relevance and included age, sex, mean arterial blood pressure, renal function and diabetes mellitus. Robust (HC3) standard errors were used.

Variable	β (m/s)	95% CI	*p* Value
Metabolic acidosis (yes vs. no)	1.41	0.87–1.95	<0.001
Recipient age (per year)	0.078	0.053–0.102	<0.001
Sex (male vs. female)	0.72	0.21–1.22	0.006
Mean arterial pressure (per mmHg)	0.008	−0.011–0.027	0.41
eGFR (per mL/min/1.73 m^2^)	−0.007	−0.020–0.005	0.25
Diabetes mellitus (yes vs. no)	1.55	0.84–2.26	<0.001

**Table 4:** Association between metabolic acidosis and frailty severity assessed by the Fried phenotype. Ordinal logistic regression was used to evaluate the association between metabolic acidosis and frailty severity (robust, pre-frail, frail). ORs (95% CIs) represent the odds of being in a higher frailty category. Model 1 was adjusted for age and sex; Model 2 was additionally adjusted for eGFR, diabetes mellitus, and log-transformed C-reactive protein. Fried components were not included as covariates.

Model	Variable	OR	95% CI	*p* Value
Model 1 (age, sex)	Metabolic acidosis (yes vs. no)	4.51	2.21–9.79	<0.001
	Recipient age (per year)	1.14	1.10–1.18	<0.001
	Sex (male vs. female)	0.94	0.49–1.80	0.850
Model 2 (fully adjusted)	Metabolic acidosis (yes vs. no)	1.83	0.78–4.43	0.167
	Recipient age (per year)	1.11	1.07–1.16	<0.001
	Sex (male vs. female)	1.12	0.57–2.24	0.738
	eGFR (per 1 mL/min/1.73 m^2^)	0.97	0.95–0.99	0.001
	Diabetes mellitus (yes vs. no)	2.51	1.25–5.04	0.010
	log(CRP)	1.56	0.76–3.30	0.232

## Data Availability

The data presented in this study are not publicly available due to privacy restrictions. Upon reasonable request, the corresponding author can provide further information about the data, subject to applicable restrictions.

## Supplementary Materials

The following supporting information can be downloaded at: https://www.mdpi.com/article/10.3390/jcm15052052/s1, Table S1: Sensitivity analysis: outcome defined as HCO3 < 22 mEq/L (Firth logistic regression), Table S2: Sensitivity analysis: outcome defined as HCO3 < 20 mEq/L (Firth logistic regression), Table S3: Sensitivity analysis: model excluding log(CRP) (Firth logistic regression), Table S4: Sensitivity analysis: model excluding RAAS blockade (Firth logistic regression), Table S5: Multivariable linear regression (robust HC3 SE) for serum bicarbonate at baseline.

## Abbreviations

The following abbreviations are used in this manuscript:

ACEi	Angiotensin-converting enzyme inhibitor
ARB	Angiotensin II receptor blocker
BMI	Body mass index
CCI	Charlson Comorbidity Index
CES-D	Center for Epidemiologic Studies Depression Scale
CKD	Chronic kidney disease
CKD-EPI	Chronic Kidney Disease Epidemiology Collaboration
CNI	Calcineurin inhibitor
CRP	C-reactive protein
DBP	Diastolic blood pressure
eGFR	Estimated glomerular filtration rate
HCO_3_^−^	Bicarbonate
HLA	Human leukocyte antigen
IPAQ	International Physical Activity Questionnaire
IQR	Interquartile range
MAP	Mean arterial pressure
OR	Odds ratio
PWV	Pulse wave velocity
RAAS	Renin–angiotensin–aldosterone system
SBP	Systolic blood pressure
SMD	Standardized mean difference
UPCR	Urine protein-to-creatinine ratio
