# Supplementary material for: Characterizing the Clinical, Vascular, and Functional Phenotype of Metabolic Acidosis in Kidney Transplantation: A Cross-Sectional Study
Source: J Clin Med. 2026 Mar 8;15(5):2052. doi: 10.3390/jcm15052052 (PMC12986342; doi:10.3390/jcm15052052)
Supplement: Supplementary file 1 [file jcm-15-02052-s001.zip › jcm-4163234-supplementary.pdf]

Supplementary Table S1. Sensitivity analysis: outcome defined as HCO3 < 22 mEq/L (Firth logistic regression)

**Variable**	**Odds Ratio (95% CI)**	**p value**
Recipient age (years)	0.96 (0.93, 0.99)	0.008
Sex: Male vs Female	0.67 (0.35, 1.27)	0.220
eGFR (mL/min/1.73 m²)	0.97 (0.95, 0.99)	<0.001
Diabetes: Yes vs No	1.98 (0.86, 4.81)	0.111
Donor age (years)	1.00 (0.97, 1.04)	0.804
Proteinuria: Detectable vs Undetectable	1.38 (0.67, 2.88)	0.384
Anti-HLA: Present vs Absent	1.53 (0.78, 3.00)	0.216
Log C-reactive protein [log(1+CRP)]	4.15 (2.16, 8.40)	<0.001
RAAS blockade: On vs Off	1.67 (0.82, 3.46)	0.158

Supplementary Table S2. Sensitivity analysis: outcome defined as HCO3 < 20 mEq/L (Firth logistic regression)

Variable	Odds Ratio (95% CI)	p value
Recipient age (years)	1.01 (0.98, 1.05)	0.582
Sex: Male vs Female	0.58 (0.29, 1.16)	0.122
eGFR (mL/min/1.73 m²)	0.97 (0.95, 0.99)	<0.001
Diabetes: Yes vs No	1.18 (0.53, 2.58)	0.677
Donor age (years)	1.04 (1.00, 1.08)	0.065
Proteinuria: Detectable vs Undetectable	1.16 (0.55, 2.40)	0.692
Anti-HLA: Present vs Absent	0.67 (0.33, 1.33)	0.256
Log C-reactive protein [log(1+CRP)]	1.42 (0.68, 2.96)	0.348
RAAS blockade: On vs Off	2.30 (1.12, 4.74)	0.023

Supplementary Table S3. Sensitivity analysis: model excluding log(CRP) (Firth logistic regression)

Variable	Odds Ratio (95% CI)	p value
Recipient age (years)	0.97 (0.94, 0.99)	0.016
Sex: Male vs Female	0.91 (0.50, 1.67)	0.772
eGFR (mL/min/1.73 m²)	0.97 (0.96, 0.99)	<0.001
Diabetes: Yes vs No	1.97 (0.92, 4.44)	0.082
Donor age (years)	1.02 (0.98, 1.05)	0.343
Proteinuria: Detectable vs Undetectable	1.51 (0.77, 3.03)	0.231
Anti-HLA: Present vs Absent	1.73 (0.93, 3.25)	0.082
RAAS blockade: On vs Off	1.71 (0.89, 3.39)	0.110

Supplementary Table S4. Sensitivity analysis: model excluding RAAS blockade (Firth logistic regression)

**Variable**	**Odds Ratio (95% CI)**	**p value**
Recipient age (years)	0.97 (0.94, 0.99)	0.017
Sex: Male vs Female	0.83 (0.44, 1.55)	0.566
eGFR (mL/min/1.73 m²)	0.98 (0.96, 0.99)	0.002
Diabetes: Yes vs No	1.70 (0.76, 3.97)	0.201
Donor age (years)	1.01 (0.98, 1.04)	0.552
Proteinuria: Detectable vs Undetectable	1.61 (0.81, 3.28)	0.176
Anti-HLA: Present vs Absent	1.64 (0.85, 3.19)	0.137

Variable	Odds Ratio (95% CI)	p value
Log C-reactive protein [log(1+CRP)]	3.55 (1.88, 7.01)	<0.001

Supplementary Table S5. Multivariable linear regression (robust HC3 SE) for serum bicarbonate at baseline

Variable	β (mmol/L)	95% CI	p value
Intercept	21.849	20.100 to 23.597	<0.001
Recipient age (years)	0.013	-0.005 to 0.030	0.160
Sex (male vs female)	0.215	-0.136 to 0.567	0.230
eGFR (mL/min/1.73 m²)	0.043	0.032 to 0.055	<0.001
Diabetes mellitus (yes vs no)	-0.328	-0.740 to 0.083	0.118
Donor age (years)	-0.017	-0.038 to 0.003	0.100
Proteinuria (detectable vs undetectable)	-0.671	-1.119 to -0.224	0.003
Anti-HLA antibodies (present vs absent)	0.030	-0.353 to 0.413	0.877
Log C-reactive protein [log(1+CRP)]	-0.858	-1.255 to -0.462	<0.001
RAAS blockade (ACEi/ARB)	-0.429	-0.806 to -0.052	0.026

Multivariable linear regression for serum bicarbonate

Model: bicarbonate_meq_l ~ age_years + sex + egfr + diabetes_any + donor_age_years + upcr_detectable + anti_hla + log_crp + raas_blockade. Robust (HC3) standard errors were used.
